# Cross‐Cutting Implementation Strategies to Integrate Hypertension Care Into HIV Care in Africa: Results From Six Country Studies in the HLB‐SIMPLe Alliance

**DOI:** 10.1002/jia2.70162

**Published:** 2026-10-08

**Authors:** Angela Aifah, Emmanuel K. Tetteh, Karla I. Galaviz, Michael E. Herce, Norberto Lumbandali, Martin Muddu, Lisa R. Hirschhorn

**Affiliations:** ^1^ Section for Global Health; Department of Population Health & Institute for Excellence in Health Equity NYU Grossman School of Medicine New York New York USA; ^2^ Bursky School of Public Health Washington University St. Louis Missouri USA; ^3^ Department of Applied Health Science Indiana University School of Public Health ‐ Bloomington Bloomington Indiana USA; ^4^ Implementation Science Department Centre for Infectious Disease Research in Zambia (CIDRZ) Lusaka Zambia; ^5^ Institute for Global Health and Infectious Diseases, University of North Carolina Chapel Hill North Carolina USA; ^6^ Instituto Nacional de Saúde Vila de Marracuene Província de Maputo Mozambique; ^7^ Makerere University Joint AIDS Program (MJAP) Makerere Uganda; ^8^ Department of Medical Social Sciences and the Havey Institute for Global Health, Feinberg School of Medicine Northwestern University Chicago Illinois USA

**Keywords:** Africa, hypertension, implementation science, implementation strategies, integration, people living with HIV

## Abstract

**Introduction:**

The National Heart, Lung and Blood Institute (NHLBI), in 2020, funded six implementation research trials in Africa (Botswana, Mozambique, Nigeria, South Africa, Uganda and Zambia) to test implementation strategies for integrating evidence‐based cardiovascular disease (CVD) care into primary care for people living with HIV. We identified cross‐site implementation strategies used by the six trials to integrate CVD care for people living with HIV in these diverse settings.

**Methods:**

Between December 2022 and September 2023, we reviewed protocols for each trial and followed up with study teams from the six studies to identify implementation strategies targeting the individual (healthcare worker [HCW] and patient), facility and system levels. Guided by the Expert Recommendations for Implementing Change (ERIC) taxonomy of implementation strategies, we grouped and defined strategies used for each study. We then specified each strategy (i.e. actor, the action, action targets, temporality, dose, implementation outcomes addressed and theoretical justification) to understand similarities and differences in how cross‐cutting implementation strategies were operationalized.

**Results:**

Ten implementation strategies were identified across the six trials for integrating hypertension (six studies) and diabetes (one study) care into existing HIV primary healthcare platforms. Three strategies, HCW training, audit and feedback, and practice facilitation or coaching, were implemented in all trials. Function and form synthesis showed that while these strategies shared the common goal of improving provider capacity and quality of care, their delivery varied across settings. HCW training was adapted in duration, frequency and trainer type; audit and feedback processes differed in how data were used and integrated with district quality improvement structures; and practice facilitation approaches ranged from study‐led to health system‐led models. These adaptations reflected contextual differences in health system capacity and available resources while maintaining a shared focus on strengthening HIV‐CVD service integration.

**Conclusions:**

With the increasing burden of CVD among people living with HIV, identifying implementation strategies for integrating CVD care into HIV primary healthcare is critical. Our study provides contextually relevant information on cross‐site implementation strategies aimed at achieving this in six African countries. The findings underscore the necessity of strategies that extend beyond conventional training, highlighting coaching, facilitation and developing data systems as central to approaches to improve integrated CVD care for people living with HIV in low‐resource settings.

## 1 Introduction

Effective antiretroviral therapy (ART) has enabled millions of people living with HIV to live longer, healthier lives [1, 2]. However, this achievement has led to an emerging challenge: the increasing burden of cardiovascular diseases (CVDs) in people living with HIV [3, 4]. Africa, where the majority of people living with HIV reside (two‐thirds) [5], is grappling with the dual burden of both HIV and CVD, such as hypertension [3, 6]. A recent systematic review and meta‐analysis of studies noted that roughly 21.9% of people living with HIV have hypertension [7]. Yet, health systems across many African countries have yet to provide care that integrates HIV management with CVD prevention, diagnosis and treatment [8, 9]. This gap in care results in poor health outcomes—and further limits the ability of this population to achieve Sustainable Development Goal 3 (Good Health and Well‐Being)—especially when rising rates of hypertension and diabetes go unrecognized and untreated within the context of existing HIV care structures [4, 10, 11, 12].

To address this challenge, the National Heart, Lung, and Blood Institute (NHLBI) and the Fogarty International Center launched the Heart, Lung, and Blood Co‐morbiditieS Implementation Models in People Living with HIV (HLB‐SIMPLe) Alliance in 2020 to support the integration of CVD care into HIV treatment. The Alliance includes six independent late‐stage implementation research trials in Botswana, Mozambique, Nigeria, South Africa, Uganda and Zambia, where research teams are testing and optimizing implementation strategies to integrate hypertension and diabetes care into routine HIV care in primary healthcare settings [13]. These strategies aim to ensure effective diagnosis, treatment, and control of hypertension and diabetes for people living with HIV, integrated within existing HIV primary healthcare centres (PHCs), using evidence‐based interventions adapted to local contexts.

As research on the integration of CVD into HIV care grows within the field of implementation science, there is an increasing focus on understanding the most effective implementation strategies in low‐resource settings, especially in Africa [14]. While much has been written about the outcomes of integrated care models [15, 16, 17], there remains a limited body of literature that identifies, describes and compares the specific implementation strategies used in diverse settings. In this manuscript, we present findings examining implementation strategies used across six HLB‐SIMPLe studies conducted in Africa. Using implementation research methods, we also examined how these strategies were designed and adapted for local contexts using a form versus function synthesis.

## 2 Methods

To identify the common implementation strategies used to integrate hypertension and diabetes screening and management into HIV care across the six studies, we drew on the expertise of the Dissemination and Implementation Science (D&I) Subcommittee of the HLB‐SIMPLe Alliance. The Alliance structure, including its working groups and subcommittees, is documented in detail elsewhere [13]. The D&I subcommittee includes at least two implementation researchers from each study team, along with members of the HLB‐SIMPLe Research Coordinating Center. These members included principal investigators, co‐investigators and study coordinators involved in protocol design and/or study implementation. A summary of the six trials, including study designs and outcomes, is provided in Supplementary Table 1.

Between December 2022 and September 2023, we reviewed protocols for each of the six trials and followed up with study teams to identify implementation strategies targeting the individual level (healthcare workers and patients), facility level and health‐system level. We reviewed protocol information available on ClinicalTrials.gov and obtained full study protocols from the respective teams to supplement registry data. Data extraction—conducted by EKT of the HLB‐SIMPLe Research Coordinating Center—focused on sections describing implementation strategies used in each intervention.

Guided by the Expert Recommendations for Implementing Change (ERIC) taxonomy [18], we grouped, defined and classified the implementation strategies identified in each study. To compare strategy use across studies, we created a matrix with the six trials as columns and implementation strategies as rows, which allowed us to identify shared strategies and estimate their frequency across sites. Strategy definitions and ERIC mapping are provided in Supplementary Table 2.

We then specified each implementation strategy using Proctor and colleagues’ reporting framework [19], including actor, action, action targets, temporality, dose, implementation outcomes addressed and theoretical justification. Because these were hybrid effectiveness‐implementation trials, we also captured relevant effectiveness outcomes along with implementation outcomes. Each study team reviewed and verified the extracted information to ensure that strategy specifications accurately reflected the original trial design.

To further compare implementation strategies across the six HLB‐SIMPLe studies, we conducted a function‐and‐form synthesis guided by Proctor's specification framework and the forms‐and‐functions framework by Perez Jolles and colleagues [19, 20]. We synthesized implementation strategies by tabulating data across all sites and identifying recurring themes across key specification domains. In this analysis, function referred to the shared purpose or intended mechanism of a strategy across studies, whereas form referred to the specific way that strategy was operationalized in each local context.

Using protocol data [21, 22, 23, 24, 25, 26] verified by site investigators (Tables 3, 4, 5), we examined the strategies implemented across all six studies. We summarized functions as concise statements describing each strategy's primary goal across studies. Forms were categorized according to key specification dimensions, including delivery actor, degree of integration with existing health‐system structures, frequency or intensity and target level. We then developed a comparative matrix to display the presence or absence of these forms across the six country trials, enabling cross‐site comparison while preserving contextual differences in implementation.

This cross‐project synthesis did not involve human participant recruitment or the collection of individual‐level data. The analysis was based on a review of approved study protocols and verification of information provided by study teams; therefore, institutional review board approval and informed consent were not required. All original implementation trials received ethical approval from their respective institutional review boards and national ethics committees in Botswana, Mozambique, Nigeria, South Africa, Uganda and Zambia prior to implementation.

## 3 Results

Table 1 presents 10 implementation strategies used across at least two of the six HLB‐SIMPLe trials. Each study used between 6 and 9 of these shared strategies to integrate hypertension care—and in one study, diabetes care as well—into HIV service delivery platforms. Three strategies were implemented in all six studies: healthcare worker training, audit and feedback, and practice facilitation and coaching. Other commonly used strategies included enabling patient education and clinic workflow modifications (five studies each), task shifting/sharing and electronic health record adaptations (four studies each), and multi‐month dispensing, treatment partners, and point‐of‐care testing in fewer studies. Because healthcare worker training, audit and feedback, and practice facilitation and coaching were the only strategies used in all six studies, we examined these in greater depth.

**TABLE 1:** Implementation strategies used at each of the six HLB‐SIMPLe research trials.

	Trial location and name						
Implementation strategy used	Botswana InterCARE	Mozambique SCALE‐SAIA	Nigeria MAP‐IT	South Africa iHEART‐SA	Uganda PULESA	Zambia TASKPEN	Total # of trials per strategy
1. Healthcare worker training	✓	✓	✓	✓	✓	✓	**6**
2. Audit and feedback	✓	✓	✓	✓	✓	✓	**6**
3. Practice facilitation and coaching	✓	✓	✓	✓	✓	✓	**6**
4. Enabling patient education	✓	•	✓	✓	✓	✓	**5**
5. Clinic workflow modifications	•	✓	✓	✓	✓	✓	**5**
6. Task shifting and sharing	•	✓	✓	✓	•	✓	**4**
7. Electronic health record systems modifications	✓	✓	•	✓	•	✓	**4**
8. Multi‐month dispensing of NCD medications	•	✓	•	•	✓	✓	**3**
9. Using treatment partners	✓	•	•	•	✓	•	**2**
10. Point‐of‐care testing (HgbA1c, lipid panel, Albumin:Cr ratio)	**•**	**•**	**•**	**•**	**•**	✓	**1**
**Total # of strategies per trial**	**6**	**7**	**6**	**7**	**7**	**9**	

*Note*: ✓ = strategy used; • = not used.

Abbreviation: NCD, non‐communicable disease.

Table 2 summarizes the shared functions and key variations in form for the three implementation strategies used across the six trials. Although healthcare worker training, audit and feedback, and practice facilitation/coaching served similar purposes (function) across studies—strengthening provider capacity, supporting data‐driven improvement and reinforcing fidelity—their delivery (form) differed by context. Variation was observed in who led implementation, the extent of integration with Ministry of Health (MOH) or district structures, the frequency and intensity of support, and the specific cadres targeted. These findings suggest that common implementation goals were maintained across studies, while operational forms were adapted to local health system realities.

**TABLE 2:** Shared functions and forms of cross‐cutting strategies across all six HLB‐SIMPLe trials.

Strategy	Shared function	Variations in form	Botswana	Mozambique	Nigeria	South Africa	Uganda	Zambia
**Healthcare worker training**	Built provider capacity to detect, treat and counsel patients with hypertension within HIV services.	Delivered jointly with MOH or district trainers	✓	✓	•	•	•	✓
		Delivered mainly by study staff or facilitators	•	•	✓	✓	✓	✓
		Included continuous or refresher sessions	✓	✓	✓	✓	✓	✓
		Targeted multiple cadres (task‐shared)	✓	✓	✓	✓	✓	✓
**Audit and feedback**	Used routine data to identify performance gaps, provide feedback and drive quality improvement.	Integrated with MOH/district QI structures	✓	✓	•	•	•	✓
		Led primarily by study/practice facilitators	•	•	✓	✓	✓	✓
		Frequent cycles (weekly–monthly)	✓	✓	✓	✓	•	✓
		Data dashboards or cascade reports used	✓	✓	✓	✓	✓	✓
**Practice facilitation/coaching**	Provided ongoing mentorship and problem‐solving to sustain fidelity and performance.	Delivered by study staff or facilitators	✓	•	✓	✓	✓	✓
		Transitioned to MOH/district‐led facilitation	•	✓	•	•	•	✓
		Continuous or on‐site support	✓	✓	✓	✓	✓	✓
		Included peer or team‐based mentoring	✓	•	•	✓	✓	•

*Note*: ✓ = form used; • = not used.

Abbreviations: MOH, Ministry of Health; QI, quality improvement.

### 3.1 Healthcare Worker Training

As shown in Table 3, healthcare worker training was used across all six studies to strengthen provider capacity for integrating hypertension care (and diabetes in one study) into HIV services. Across settings, training focused on improving knowledge and skills related to screening, diagnosis, treatment, counselling and documentation, while also preparing providers to work within task shifted/shared models of care. Training generally occurred at study initiation and was reinforced through refresher or ongoing sessions during implementation.

**TABLE 3:** Healthcare worker training: cross‐site implementation strategy specified using Proctor and colleagues’ reporting framework.

Trial	Actor (who is delivering?)	Action (what is being done)	Target (who receives?)	Temporality (when?)	Dose (duration and frequency)	Implementation and effectiveness outcomes targeted	Justification (mechanism)
**InterCARE (**Botswana)	Two master trainers from MOHW facilities under guidance of NCD programme officer and research team	A master curriculum will be created and implemented focused on hypertension screening, diagnosis, management (medication and counselling) and CVD risk management Two master trainers will train prescribers (doctors, specialists, family nurse practitioners) in CVD risk management Virtual training was also made available to all prescribers	Doctors and specialist RN prescribers (family nurse practitioners)	Training at the start of the study with refresher training as needed (for new prescribers to the clinics)		Primary: effectiveness; Secondary: acceptability; Additional: design and implementation of a curriculum that is patient‐focused	A barrier to guideline‐based clinical care in hypertension is limited continued professional development (CPD) opportunities
**SCALE‐SAIA HTN** (Mozambique)	Research team	At the initiation of each intensive wave, district MOH supervisors and health facility staff receive a 3‐day training on the SAIA‐HTN implementation strategy and operational processes, and to ensure adequate SAIA‐HTN knowledge and uptake. Also reinforcement of clinical skills for providers on MOH‐mandated clinical management of HTN.	District and health facility‐level providers and staff	At study start and afterwards as needed (new staff or changes to MOH guidelines)	Three days for initial training.	Acceptability, adoption, effectiveness, reach	
**MAP‐IT** (Nigeria)	Research team	The community nurses trained on identifying, counselling, treating and referring (ICTR) adult people living with HIV with HTN using the “5 As” counselling strategy (Ask, Assess, Advise, Assist and Arrange). Practice facilitators (PFs), who are retired community nurses, were be trained to coach the community nurses on the “3Es”: Engaging, Enhancing and Evaluating their tasks.	PFs, nurses, case managers	Throughout study	Every 3 months	Effectiveness, reach	Capacity‐building specifically on Task Shifting Strategy for Hypertension (TASSH) for the community nurses.
**IHEART‐SA** (South Africa)	Research team	Ongoing training sessions for healthcare workers delivered by the care coordinator on elevated blood pressure detection and treatment, data interpretation, and patient−provider communication skills.	HCW	Throughout intervention	Monthly (number of sessions dependent on availability of the staff)	Guideline fidelity	Psychological capability
**PULESA** (Uganda)	Research team	Three types of training are offered in PULESA‐Uganda intervention arms to provide in‐depth discussion of integrated HIV‐HTN care management: (1) One‐time in‐person basic training (HTN BASIC and PLUS); (2) Follow‐up training on a quarterly and ad hoc basis, primarily through the case management forum described above (HTN‐PLUS); (3) Occasional 1:1 clinician consultation with the research nurse (HTN PLUS).	Providers; doctors, clinical officers, nurses and HIV peer counsellors	Start, throughout intervention and as needed		Effectiveness, reach	Training will enhance the capacity of healthcare provider to provide better integrated HIV and hypertension care to people living with HIV and hypertension
**TASKPEN** (Zambia)	Research team	Provide initial and refresher training on WHO PEN adapted for non‐physician health worker‐led management of hypertension, diabetes and dyslipidaemia in Zambia, with clear recommendations for when to consult or refer patients to specialist physician and higher‐level facilities.	Providers; doctors, clinical officers, nurses, pharmacists, laboratory technicians, data staff and community health workers	Prior to intervention start and periodic refresher trainings thereafter as needed	Five‐day initial one‐time training; 2‐day periodic refresher training (typically once after initial training)	Effectiveness, reach, fidelity, adoption, acceptability	Capacity‐building on integrated cardiometabolic NCD care, creating capabilities to enable task‐shifting and task‐sharing

Abbreviations: CVD, cardiovascular disease; HCW, healthcare worker; HTN, hypertension; MOH, Ministry of Health; NCD, non‐communicable disease; PF, practice facilitator; RN, registered nurse.

Although the function of training was consistent across studies, its form varied by context. Differences were observed in who delivered the training, the duration and frequency of sessions, and the cadres targeted. In some studies (e.g. Botswana, Mozambique, Zambia), training was co‐delivered with MOH or district personnel, whereas in others (e.g. Nigeria, South Africa, Uganda), it was led primarily by research teams or facilitators. Despite these differences, training was consistently intended to support adoption, fidelity, reach and provider capability for integrated care.

### 3.2 Audit and Feedback

Table 4 shows that audit and feedback was used in all six trials as a strategy to promote data‐informed implementation and continuous quality improvement. Across studies, teams reviewed routine service or study data to assess performance, identify implementation gaps and guide corrective action. Common feedback mechanisms included dashboards, cascade reports and structured review meetings focused on improving delivery of integrated care.

**TABLE 4:** Audit and feedback: cross‐site implementation strategy specified using Proctor and colleagues’ reporting framework.

Trial	Actor (who is delivering?)	Action (what is being done)	Target (who receives?)	Temporality (when?)	Dose (how often)	Implementation and effectiveness outcome targeted	Justification (mechanism)
**InterCARE (**Botswana)	Senior study staff	Review of data (enrolment, retention, BP control) to identify any barriers and improve on the quality of the delivery and study implementation	Clinic managers and staff	Throughout intervention	Every week	Primary: effectiveness Secondary: unintended side effects	Site assessments help improve processes and implementation in real‐time throughout the course of the study
**SCALE‐SAIA HTN** (Mozambique)	Research team during intensive phase, MOH district supervisor during maintenance phase	Using the results of the first two components of SAIA (cascade analysis tool and process mapping), a micro‐intervention targeting a specific cascade analysis step and/or service bottleneck is proposed. The micro‐intervention is operationalized in terms of its goal, scope, timeframe and specific tasks. Micro‐interventions are implemented and their impact assessed through a plan‐do‐study‐act cycle. The fidelity of implementation is assessed, and the decision is made to adopt, adapt or abandon the micro‐intervention.	Health facility staff and managers	Implemented once after first two components of SAIA have identified potential targets for QI specific to the implementing unit.	Typically monthly	Fidelity, reach, effectiveness, adoption, maintenance, appropriateness	
**MAP‐IT** (Nigeria)	Practice facilitators and research team	− Onsite meetings with the PFs twice a month for the first month and then monthly thereafter to facilitate practice changes to support the implementation of TASSH at the PHC. − Fidelity tools measuring dose, adherence, differentiation and responsiveness are administered to PFs, nurses and/or people living with HIV to determine implementation fidelity of TASSH and PF.	Community nurses and PFs	Throughout intervention	Bi‐monthly then monthly	Primary: fidelity, reach, effectiveness, adoption, maintenance. Secondary: acceptability, feasibility	To ensure adherence to study protocol and make adaptations as necessary.
**IHEART‐SA** (South Africa)	Care coordinator	Sessions delivered by the care coordinator where the dashboard data for a given month is presented to healthcare workers, clinic managers and staff. Programmatic feedback is provided to discuss how to improve case detection and treatment.	HCW	Throughout intervention	Monthly	Guideline fidelity	Reflective motivation
**PULESA** (Uganda)	Research nurse; study coordinator	On a quarterly basis, the research team compiles a report for each HTN‐PLUS clinic that describes their hypertension cascade metrics. Clinics are encouraged to schedule a meeting with the research team to discuss ideas for improving their cascade metrics.	Providers; doctors, clinical officers, nurses and HIV peer counsellors	Throughout intervention	Quarterly	Primary: fidelity, reach, effectiveness, adoption, maintenance; Secondary: acceptability, feasibility	Enables the clinic team to appreciate their efforts to integrate HIV‐HTN care, identify challenges that hamper integration and devise mechanism to improve performance. Allows utilization of data to improve performance. Easier to implement and requires less resources while highlighting hypertension as a priority comorbidity in HIV services
**TASKPEN** (Zambia)	Research team	Regular collaborative data review meetings among frontline health workers focused on dashboard indicators to identify and act on areas for quality improvement. Indicators are care cascade‐focused and prioritize screening, diagnosis, and treatment initiation for hypertension, diabetes and hyperlipidaemia.	Providers; doctors, clinical officers, nurses, pharmacists, laboratory technicians, data staff and community health workers	Throughout intervention	Monthly	Fidelity, reach, effectiveness, adoption, maintenance	Promote health worker engagement in the intervention and data use for quality improvement.

Abbreviations: HCW, healthcare worker; HTN, hypertension; MOH, Ministry of Health; NCD, non‐communicable disease; PF, practice facilitator; PHC, primary healthcare centre.

The specific form of audit and feedback differed across settings. Variation was seen in who led the process, how frequently feedback was provided and the degree of integration with existing health system quality improvement structures. Some studies (e.g. Nigeria) used more intensive feedback cycles early in implementation before shifting to less frequent review, while others (e.g. Mozambique, South Africa, Uganda, Zambia) maintained monthly or quarterly feedback throughout. Across all studies, the strategy was intended to strengthen fidelity and effectiveness, while in some settings supporting adoption, maintenance, feasibility and acceptability.

### 3.3 Practice Facilitation and Coaching

Table 5 summarizes practice facilitation and coaching, which was also implemented across all six trials to provide ongoing support for integrated service delivery. In all settings, this strategy involved mentorship, supervision and problem‐solving to help providers apply training in routine practice, address challenges and sustain implementation over time. Facilitation often complemented training and audit and feedback by reinforcing skills and supporting adaptation within clinic workflows.

**TABLE 5:** Practice facilitation or coaching: cross‐site implementation strategy specified using Proctor and colleagues’ reporting framework.

Trial	Actor (who is delivering?)	Action (what is being done)	Target (who receives?)	Temporality (when?)	Dose (how often)	Implementation and effectiveness outcome targeted	Justification (mechanism)
**InterCARE** (Botswana)	Study nurses	On‐site coaching that includes face‐to‐face observation	Providers	Throughout intervention	Monthly study site visits and/or as needed	Primary: fidelity, feasibility	Maintain knowledge gained from initial provider training
**SCALE‐SAIA HTN** (Mozambique)	Research team during intensive phase, MOH district supervisor during maintenance phase	A convened group of frontline service providers is led by an external facilitator (with expertise in SAIA) to facilitate the process of going through the SAIA multi‐component implementation strategy. These SAIA‐strategy meetings usually occur monthly and the research team members with SAIA expertise provide ongoing support and/or feedback on SAIA implementation to the service delivery team initially and then this work is taken over by MOH district supervisors	Health facility staff and managers	Implemented as overarching step of the SAIA intervention	Typically, monthly	Fidelity, reach, effectiveness, adoption, maintenance, appropriateness	
**MAP‐IT** (Nigeria)	Practice facilitators	PFs provided support for nurses during the 12‐month intervention, assisting with issues and addressing barriers to implementation of TASSH in the PHCs. PFs provide support to the nurses using the “3 E” strategy: Engaging, Enhancing and Evaluating their tasks.	Community nurses	Throughout intervention	Throughout intervention; continuous	Primary: fidelity, reach, effectiveness, adoption, maintenance. Secondary: acceptability, feasibility	To leverage the existing healthcare capacity of Nigeria (by including retired trained nurses) and capacity‐building of current community nurses.
**PULESA** (Uganda)	Research nurse	A study nurse, skilled in the management of hypertension, will be available by phone to provide 1:1 consultations to providers and facilitate referrals to experts at the Uganda Heart Institute or other appropriate facilities for difficult or refractory cases.	Providers	Throughout intervention	Continuous as needed	Primary: fidelity, reach, effectiveness, adoption, maintenance; Secondary: acceptability, feasibility	This is part of hypertension training strategy
**TASKPEN** (Zambia)	Research team; MOH	CIDRZ/TASKPEN Mentors observe and coach MOH providers, with a focus on nurses, CHWs/peers, and pharmacy staff, to assume NCD care responsibilities. CIDRZ/TASKPEN Mentors and MOH HIV/NCD Integration Champions engage in interactive problem‐solving and support basic quality improvement activities that engage the entire enhances integrated care delivery during visits.	Providers; doctors, clinical officers, nurses, pharmacists, laboratory technicians, data staff and community health workers	Throughout intervention	Twice weekly to start; then once weekly once proficient	Fidelity, reach, effectiveness, adoption, maintenance	Empathically build skills among health providers to use TASKPEN intervention in their practice environment.
**iHEART‐SA (**South Africa)	Care coordinator	Care coordinator recruited and trained to assist healthcare workers with implementation of the elevated blood pressure detection and treatment protocol. Ongoing training sessions for healthcare workers delivered by the care coordinator on elevated blood pressure detection and treatment, data interpretation, and patient−provider communication skills.	Healthcare workers	Throughout active intervention period	Ongoing	Guideline fidelity	Social opportunity

Abbreviations: CHW, community health worker; HCW, healthcare worker; HTN, hypertension; MOH, Ministry of Health; NCD, non‐communicable disease; PF, practice facilitator.

As with the other cross‐cutting strategies, the form of facilitation/coaching varied considerably by study. Support was delivered by different actors, including study nurses, practice facilitators, care coordinators, MOH supervisors and internal facility champions. The intensity of support ranged from continuous or as‐needed consultation to structured weekly or monthly engagement. Despite this variation, practice facilitation/coaching consistently aimed to strengthen fidelity, adoption and maintenance of integrated care, while also building local capacity for sustained implementation.

## 4 Discussion

This study identifies and specifies implementation strategies used within and across six studies within the HLB‐SIMPLe Alliance, aimed at integrating hypertension and diabetes (one study only) care into HIV service delivery platforms in Africa. Ten implementation strategies were used in at least two or more studies to facilitate this integration. All studies used multiple strategies, with healthcare worker training, audit and feedback, and practice facilitation and coaching emerging as the three strategies used across all six studies. The way these strategies were implemented, along with other site‐specific strategies, reflect the diverse and context‐sensitive approaches necessary to enhance the integrated management of hypertension within routine HIV care.

While strategies such as healthcare worker training, audit and feedback, and practice facilitation are widely used to promote healthcare service integration, the way integration is achieved differs fundamentally between high‐ and low‐resource settings. In high‐income countries, robust infrastructure, digital health systems and specialist support can allow patients to act as the integrators of their own services, navigating and coordinating care across multiple providers [27, 28, 29]. In contrast, in low‐resource settings, weak system infrastructure and workforce shortages often place the burden of integration on healthcare workers themselves. To make integration work, they often assume multiple roles and responsibilities alongside their routine tasks, highlighting both the ingenuity and strain involved in adapting existing platforms to deliver new services [27, 30, 31].

The capacity challenge low‐resource settings face underscores the importance of continuous, hands‐on support beyond one‐time training sessions for healthcare workers at the centre of integrating care. While initial training was universal across the trials, capacity‐building extended beyond these training events. This approach acknowledges that training alone is insufficient for sustained individual and system practice change [32, 33]. Instead, these studies deployed practice facilitation strategies to help healthcare workers navigate real‐world challenges, utilize performance data, address implementation barriers and develop skills to provide the protocol‐defined hypertension diagnosis and management in their specific practice contexts. The variation in how this strategy was implemented, including who acted as facilitators (e.g. retired nurses, district supervisors, research teams), facilitation frequency across trials and the specific actions taken, reflected adaptation to factors in the implementation context as well as availability of dedicated trial funding. Understanding these variations is essential for explaining differences in outcomes across studies.

Another key finding was the focus on data‐driven monitoring and decision‐making through audit and feedback. Across the trials, teams used programme and study data to inform practice, track adherence to protocols and refine or strengthen strategies in real‐time to address identified gaps. This approach aligns with global trends in implementation science, where audit and feedback is recognized as a cornerstone of effective healthcare interventions [32]. However, the frequency and intensity of feedback varied. Some trials provided ongoing, iterative feedback, while others relied on monthly or quarterly reviews. These differences highlight the need to balance timely course correction with the practical constraints of resource‐limited settings, while also addressing the persistent challenge of obtaining and cleaning data to make it usable for facilitation and feedback processes.

This study also leveraged the power of cross‐study analysis and the value of specifying implementation strategies in detail. Even when similar strategies were used across studies, contextual variation shaped how they operated. While overall strategy selection reflected the specific barriers and facilitators in each setting [21, 22, 23, 24, 25, 26], and justification was context‐dependent rather than uniform, the shared strategies also reflected common function despite differences across the six countries [19, 34, 35]. While there were common strategies across the different trials, differences in how they were operationalized (i.e. the form) reinforce the need for clear articulation of design mechanisms and contextual drivers to enhance replicability and inform adaptation across new settings [34]. These findings also highlight the value of reciprocal innovation, where lessons from low‐resource settings inform practice in high‐income countries. Workforce‐oriented strategies such as facilitation, coaching and pragmatic approaches to audit and feedback may be particularly relevant for high‐need communities in the United States, including among people living with HIV.

More broadly, these findings contribute to the growing literature on health service integration in low‐resource settings, illustrating how cross‐cutting strategies, particularly those that are relatively feasible, cost‐effective and workforce‐oriented, such as healthcare worker training integrated with supportive supervision and task shifting and use of routine health system data to monitor and strengthen care [36, 37], can support the integration of CVD and HIV services. As health systems worldwide seek to optimize care delivery and realize efficiencies at scale, understanding how to integrate services effectively, whether for hypertension, HIV or other chronic conditions, is increasingly relevant [31, 38]. While care integration remains a priority across different settings, how it is done, and what implementation strategies are most relevant, differs significantly based on workforce capacity, infrastructure and resource availability [27]. Our findings highlight the importance of capacity‐building beyond training, emphasizing coaching and facilitation as essential for sustaining practice change in healthcare delivery. We also found the vital role of audit and feedback in data‐driven decision‐making, demonstrating how continuous quality improvement can strengthen implementation efforts.

Several limitations should be acknowledged. First, the analysis was based on reported strategies from study protocols and team self‐reports, which may not fully capture real‐time adaptations. Second, while we identified cross‐cutting strategies, we did not assess their fidelity or their impact on implementation or patient outcomes. The effectiveness of the strategies is still emerging as the six studies end and final analyses are underway. Future research should evaluate which strategies yield the greatest impact and how they operate mechanistically in practice. Finally, as these studies were situated in diverse African contexts, health system differences, resource availability and policy environments may limit the transportability of findings to other regions.

## 5 Conclusions

This study provides an overview of cross‐cutting implementation strategies used to integrate hypertension and diabetes care into HIV care across six African countries in the HLB‐SIMPLe Alliance. Healthcare worker training, audit and feedback, and practice facilitation were common strategies across studies, all focused on strengthening workforce capacity and supporting fidelity to evidence‐based interventions. By specifying and identifying these strategies, this work adds to the knowledge base on scaling integrated care models in resource‐limited settings. In particular, the relatively low‐cost and adaptable nature of these strategies may explain their consistent use across diverse contexts. These findings can inform future efforts to integrate CVD and HIV care in Africa and beyond, helping guide health system strengthening and policy development in regions facing similar challenges. Moving forward, research should assess the comparative effectiveness of these strategies, clarify their mechanisms and examine additional approaches to enhance patient engagement and ensure long‐term sustainability.

## Author Contributions

AA and EKT contributed equally as co‐first authors. AA, EKT and LRH conceptualized the study. AA, KIG, MEH, NL, MM and LRH led site‐level study implementation and data collection. EKT conducted data synthesis and contributed to drafting the manuscript. LRH provided overall guidance, methodological expertise and critical revisions. All authors (AA, EKT, KIG, MEH, NL, MM, LRH) contributed to interpretation of findings, reviewed manuscript drafts and approved the final version for submission.

## Funding

The development of this manuscript was supported by the United States of America National Institute of Health (NIH) via the National Heart, Lung and Blood Institute (NHLBI), including Heart, Lung, and Blood Co‐morbiditieS Implementation Models in People Living with HIV (HLB SIMPLe) Alliance Awards. This study was supported by Research Coordinating Center Grant Award: U24HL154426, Botswana InterCARE Trial: UG3HL154499, Mozambique SCALE SAIA‐HTN Trial: UH3HL156390, R01HL142412 and P30AI027757, Nigeria MAP‐IT Trial: UH3HL154498, South Africa iHEART‐SA Trial: UH3HL156388, Uganda PULESA Trial: UH3HL154501, and Zambia TASKPEN Trial: UH3HL156389. Angela Aifah is supported by an NHLBI‐funded career award grant #HL165086. Karla I. Galaviz is supported by an NHLBI‐funded award grant #R03HL174207.

## Disclaimer

The content is solely the responsibility of the authors and does not necessarily represent the official views of the National Heart, Lung, and Blood Institute, the Fogarty International Center, the National Institutes of Health or the United States Department of Health and Human Services. The funder had no role in the conceptualization of the study, design of data collection tools or analyses, or the decision to publish.

## Conflicts of Interest

The authors declare no conflicts of interest.

## Supporting information




**Supplementary Table 1**: HLB‐SIMPLe Alliance Countries, Research Trials, and Trial Registrations. **Supplementary Table 2**: HLB‐SIMPLe Implementation Strategies, Their ERIC Strategy Mapping, and Definitions

## Data Availability

All data generated or analysed during this study are included in this published article and its Supplementary Information Files.
