# Supplementary material for: Cross‐Cutting Implementation Strategies to Integrate Hypertension Care Into HIV Care in Africa: Results From Six Country Studies in the HLB‐SIMPLe Alliance
Source: J Int AIDS Soc. 2026 Oct 8;29(Suppl 5):e70162. doi: 10.1002/jia2.70162 (PMC13650416; doi:10.1002/jia2.70162)
Supplement: Supplementary file 1 [file JIA2-29-e70162-s001.docx]

**APPENDIX**

| **Supplementary Table 1.** HLB-SIMPLe Alliance Countries, Research Trials, and Trial Registrations. | | | |
| --- | --- | --- | --- |
| **Trial Registration Number** | **Country – Trial Name** | **Study Design** | **Study Outcome Measures** |
| NCT05414526 | Botswana –  Integrating Hypertension and Cardiovascular Disease Care into Existing HIV Service Package in Botswana (InterCARE) | Cluster Randomized Control Trial: 14 clinics; 7 receive InterCARE, 7 standard care. | Primary: RE-AIM assessment (reach, effectiveness, adoption, implementation fidelity and maintenance)  Secondary: Implementation feasibility, acceptability, CVD risk factor % change |
| NCT05002322 | Mozambique –  Scaling Out and Scaling Up the Systems Analysis and Improvement Approach to Optimize the Hypertension Diagnosis and Care Cascade for HIV-infected Individuals (SCALE-SAIA HTN) | Stepped wedge design: Randomized 9-month waves across 6 districts in Maputo, Mozambique. | Effectiveness outcomes:  *Individual clinical* (controlled HTN and viral load suppression)  *Service-level* (HTN screening, diagnosis, treatment and medication adherence)  Other outcomes (RE-AIM): reach, adoption, implementation, maintenance, and cost-effectiveness |
| NCT05031819 | Nigeria –  Managing Hypertension Among People Living with HIV: an Integrated Model (MAP-IT) | Stepped wedge cluster RCT: 30 primary healthcare centers. | Primary: Adoption of task-strengthening program (TASSH)  Secondary: BP control, rate of adoption and implementation fidelity of TASSH |
| NCT05846503 | South Africa –  Integrating HIV and Heart Health in South Africa (iHEART-SA) | Type 2 hybrid stepped wedge trial: 9 primary healthcare centers. | Primary: Difference in mean systolic BP between intervention and control groups  Secondary: Implementation fidelity, adoption, maintenance, satisfaction, cost. |
| NCT05609513 | Uganda –  Strengthening the Blood Pressure Care and Treatment Cascade for Ugandans Living with HIV – Implementation Strategies to Save Lives (PULESA) | Stepped wedge cluster RCT: 16 clinics randomized to HTN-BASIC or HTN-PLUS. | Primary: BP control (% with BP<140/90)  Secondary: Scalability, sustainability, cost-effectiveness |
| NCT05005130; NCT05950919 | Zambia –  Task-shifted Adaptation of the WHO-PEN Intervention to Address Cardio-metabolic Complications in People Living with HIV in Zambia (TASKPEN) | Type II hybrid stepped wedge trial: 12 sites. | Primary: Proportion of participants with both HIV viral suppression and control of hypertension, diabetes and tobacco use.  Secondary: Implementation fidelity, adoption, reach, cost-effectiveness, acceptability, etc. |
| *^a^Table adapted with permission from Tetteh et. al., Imp Sci Comm, 2024* | | | |

| **Supplementary Table 2**. HLB-SIMPLe Implementation Strategies, Their ERIC Strategy Mapping, and Definitions | | |
| --- | --- | --- |
| **HLB-SIMPLe Implementation Strategy** | **ERIC Strategy** | **ERIC Strategy Definition** |
| 1. Healthcare Worker(s) Training | Conduct ongoing training | Plan for and conduct training in the clinical innovation in an ongoing way |
|  | Develop educational materials | Develop and format manuals, toolkits, and other supporting materials in ways that make it easier for stakeholders to learn about the innovation and for clinicians to learn how to deliver the clinical innovation |
| 2. Audit and Feedback. Continuous Quality Improvement | Audit and provide feedback | Collect and summarize clinical performance data over a specified time period and give it to clinicians and administrators to monitor, evaluate, and modify provider behavior |
|  | Facilitate relay of clinical data to providers | Provide as close to real-time data as possible about key measures of process/outcomes using integrated modes/channels of communication in a way that promotes use of the targeted innovation |
|  | Organize clinician implementation team meetings | Develop and support teams of clinicians who are implementing the innovation and give them protected time to reflect on the implementation effort, share lessons learned, and support one another’s learning |
| 3. Practice Facilitation and Coaching | Facilitation | A process of interactive problem solving and support that occurs in a context of a recognized need for improvement and a supportive interpersonal relationship |
|  | Provide clinical supervision | Provide clinicians with ongoing supervision focusing on the innovation. Provide training for clinical supervisors who will supervise clinicians who provide the innovation |
|  | Use train-the-trainer strategies | Train designated clinicians or organizations to train others in the clinical innovation |
| 4. Enabling Patient Education | Conduct educational meetings | Hold meetings targeted toward different stakeholder groups (e.g., community, patient/consumer) to teach them about the clinical innovation |
|  | Distribute educational materials | Distribute educational materials (including guidelines, manuals, and toolkits) in person, by mail, and/or electronically |
| 5. Clinic Workflow Modifications | Change physical structure, equipment and service sites | Evaluate current configurations and adapt, as needed, the physical structure and/or equipment (e.g., changing the layout of a room, adding equipment) to best accommodate the targeted innovation |
| 6. Task Shifting and Sharing | Revise professional roles | Shift and revise roles among professionals who provide care, and redesign job characteristics |
| 7. Electronic Health Record Systems Modifications | Use data warehousing techniques | Integrate clinical records across facilities and organizations to facilitate implementation across systems |
|  | Remind clinicians | Develop reminder systems designed to help clinicians to recall information and/or prompt them to use the clinical innovation |
| 8. Multi-Month Dispensing of Medications | Promote adaptability | Identify the ways a clinical innovation can be tailored to meet local needs and clarify which elements of the innovation must be maintained to preserve fidelity |
| 9. Using Treatment Partners | Intervene with patients/ consumers to enhance uptake and Adherence | Develop strategies with patients to encourage and problem solve around adherence |
|  | Involve patients/consumers and family members | Engage or include patients/consumers and families in the implementation effort |
| 10. Point-Of-Care Testing and Blood Pressure Equipment Supply | Change service sites | Change the location of clinical service sites to increase access |
|  | Change physical structure and equipment | Evaluate current configurations and adapt, as needed, the physical structure and/or equipment (e.g., changing the layout of a room, adding equipment) to best accommodate the targeted innovation |
